# Impact of COVID-19 infections on hemodialysis patients in the province of Blida, Algeria

**DOI:** 10.11604/pamj.supp.2020.37.1.26850

**Published:** 2020-12-22

**Authors:** Mourad Kastali, Ahmed Youssef Kada, Sonia Ounnas

**Affiliations:** 1 University of Blida, Department of Nephrology, Hospital Establishment Specializing in Organ and Tissue Transplantation (TOT) of Blida, Blida, Algeria,; 2 Department of Anesthesiology, University Hospital Center, Blida, Algeria,; 3 University of Blida, Department of Medical Laboratory Center, Hospital Establishment Specializing in Organ and Tissue Transplantation (TOT) of Blida, Blida, Algeria

**Keywords:** Hemodialysis, COVID-19, treatments, vulnerability, Blida

## Abstract

Patients on hemodialysis are a group at risk for infection with SARS-CoV-2 due to impaired immunity. Our knowledge of the specific characteristics of the disease in this population is limited. Our study aims to improve our understanding of diagnostic, therapeutic, and prognostic aspects of this disease. Seventy hemodialysis patients tested by RT-PCR positive for SARS-CoV-2 were hospitalized in the nephrology department from April 1 to September 30, 2020. The patients' average age was 60.3 ± 15.0 years (18 - 88 years); including 39 patients (55.7%) were male. The comorbidities found were hypertension (67.1%), diabetes (32.9%), and obesity (17.1%). Our patients' laboratory abnormalities were leukopenia (15.7%) and lymphopenia in 60% of patients. The pulmonary involvement on computed tomography was classified as moderate (74.3%) and severe in 24.3% of cases. Fifty-seven patients (78.6%) had received hydroxychloroquine and 14 patients (20%) antivirals therapy. We recorded 82.9% of cured patients and 17.1% of deaths in our series. The death occurred 8 ± 7.5 days (1 - 25 days) of hospitalization. Hemodialysis centres are high-risk places, and hemodialysis patients are considered a vulnerable population during the COVID-19 pandemic. They should be given special attention.

## Introduction

In December 2019, a pneumonia epidemic broke out in Hubei province in China due to a new coronavirus [1], called SARS-CoV-2 by the coronavirus working group of the International Committee of Viruses [2]. The infection had spread rapidly in several countries; the World Health Organization (WHO) declared it a pandemic in March 2020. The elderly or patients with comorbidities were more vulnerable to COVID-19, and the incidence of severe cases and the risk of death were high according to the various epidemiological surveys carried out. The impact of the SARS-CoV-2 virus on hemodialysis patients with an impaired immune function who are on dialysis and sometimes transported in groups is not clear [3]. Once infected, these patients are a mobile source of new contagions within the group at risk. Our knowledge of the specific characteristics of the disease in this population is limited, as only isolated observations or small series of cases on the prevalence and death rate have been reported [4].

## Methods

That single-centre prospective study carried out at the specialized hospital of organ and tissue transplantation (TOT) of Blida, Algeria during the period from Mar 13, the date of the first patient received at the department of nephrology level to Sept 30, 2020, the discharge date of the last hemodialysis patient treated for infection with SARS-CoV-2. Our hemodialysis unit is the only public structure in the province of Blida; therefore, it drains all patients requiring inpatient dialysis sessions in the various departments dedicated to the management of COVID-19.

**Inclusion and exclusion criteria:** all chronic renal failure patients on hemodialysis for at least three months treated in the centre during the study period; patients presenting with fever or respiratory symptomatology who have pulmonary CT scan suggestive of COVID-19, whose diagnosis of infection is confirmed by a positive RT-PCR (reverse transcription polymerase chain reaction). Excluded were patients with chronic renal failure not on dialysis, children under 18 years, and patients with negative RT-PCR.

Hemodialysis patients presenting with symptoms suggesting an infection due to SARS-CoV-2 referred to our hospital. A chest CT is performed. The evocative images, although not specific are: ground glass opacities, which can be of unilateral or bilateral, peripheral or multilobar distribution [5], at a late stage these ground glass opacities have a crazy paving appearance and can be pure or associated with condensations [6]. The extent of the lesions was analyzed and classified as minimal (<10%), moderate (10-25%), extensive (26-50%), severe (51 to 75%) and critical (> 75%) [7]. Confirmation of SARS-CoV-2 was performed by (RT-PCR) after taking a nasopharyngeal swab [8]. A biological assessment was performed on admission of the patient: blood count, TCK; C-reactive protein; renal assessment: blood urea, serum creatinine; serum electrolytes; dosage of glycemia, bilirubin, aspartate aminotransferase (AST), alanine- aminotransferase (ALT); D-dimer. The prescribed treatment was recommended by our supervisory authority [9]: i) hydroxychloroquine: 400 mg per day for ten days; ii) azithromycin tab: 500 mg on the first day followed by 250 mg per day for the next four days. The alternative treatment will involve: lopinavir/ritonavir: (tablet 200/50 mg) at a rate of 400 mg per day for 7 days

**Statistical analysis:** it was performed using SPSS 23 software. Qualitative variables are presented with their frequency distribution. Their mean and standard deviation presents quantitative variables. The association between qualitative variables was assessed with the chi-square test or fisher's exact test. Quantitative variables were analyzed using student's t-test. Univariate and multivariate logistic regression methods were used to explore risk factors associated with death. Statistical significance was considered to be a two-tailed p-value <0.05.

## Results

Seventy hemodialysis patients were hospitalized in our establishment for novel coronavirus disease 2019 from April 1^st^ to September 30^th^, 2020. Their mean age was 60.3 ± 15.0 years (18 - 88 years), of which 39 patients (55.7%) were men. Twelve patients (17.1%) were obese (BMI> 30Kg/m^2^). The comorbidities found were hypertension (67.1%), diabetes (32.9%), cardiovascular diseases (9.7%), chronic obstructive lung disease (7.1%), history of cancer (7.1%), and systemic disease in 4.2% of cases (Table 1). The laboratory abnormalities observed in our patients were leukopenia (15.7%), higher white blood cells count (13.7%), and lymphopenia in 60% of patients. Lung abnormalities on chest CT were ground glass opacities, crazy paving with or not condensation. This involvement was classified as mild (1.4%), moderate (74.3%), and severe in 24.3% of cases. Other images have been associated with unilateral pleural effusion (2.7%), bilateral (5.5%), and pericardial effusion, sequelae of pulmonary tuberculosis or chronic obstructive lung disease in one patient, respectively. Fifty-seven patients (78.6%) had received hydroxychloroquine, 14 patients (20%) antivirals therapy, including (04 patients) as second-line after developing signs of intolerance to hydroxychloroquine. One patient (1.4%) received only azithromycin because of minimal pulmonary involvement on CT. Fifty-eight patients (82.9%) were cured, a worsening of the general condition was observed in 12 patients (17.1%) (Table 2): i) six patients (8.5%) presented with desaturation (SPO2 <70%) under 12 liters of oxygen, were evacuated to the intensive care unit; ii) six patients (8.5%) had presented a worsening of their condition (one patient had a stroke, one patient a gastrointestinal haemorrhage, two patients had to were rehospitalized after their discharge for acute respiratory failure, and two diabetic patients presented a worsening of cardiac abnormalities already present. Unfortunately, 12 patients (17.1%) have died in our series. The death occurred 8 ± 7.5 days (1 - 25 days) of their hospitalization. In univariate analysis: diabetes (p = 0.05); dyspnea (p = 0.005), impaired consciousness (p = 0.00004) and hospitalization in an intensive care unit (0.007) were statistically significant. In multivariate analysis: dyspnea (p = 0.02), severe involvement on CT (p = 0.02) and diabetes (p = 0.01) were found to be statistically significant.

**Table 1:** patient characteristics

Parameters	N= 70	%
Age (years)	60.3 ± 15.0 (18 - 88)	
Male	39	55.7%
**Comorbidities**		
Diabetes	23	32.9%
Hypertension	47	67.1%
Cardiovascular diseases	07	9.7%
Obesity	12	17.1%
Chronic obstructive lung disease	05	7.1%
History of cancer	05	7.1%
Systemic disease	03	4.2%
**Thoracic CT scan**		
Mild	1	1.4%
Moderate	52	74.3%
Severe	17	24.3%
**Laboratory data**		
White cells count (x10^-9^/liter)	6.2 ± 3.0 (2 - 15.5)	
Lymphocyte count(x10^-9^/liter)	1.5 ± 0.5 (0.5 - 2.6)	
Aspartate aminotransferase (U/ liter)	35.1 ± 40.5 (10 - 281)	
Alanine aminotransferase (U/ liter)	25.5 ± 31.7 (4 - 178)	
**Treatment**		
Hydroxychloroquine	55	78.6%
Antiviral therapy	14	20%
Azithromycin	1	1.4%
Mortality	12	17.1%
Number of hemodialysis sessions	5.4 ± 3.1 (1 - 13)	

**Table 2:** comparison between survivors and deceased patients

Parameters.	Nonsurvivors (N =12)	Survivors (N = 58)	P-value
Average age (years)	66.3 ± 15.8	59.1 ± 14.6	0.6
**Influence of age**			0.1
< 70 years	6 (12.3%)	43 (87.8%)	
≥ 70 years	6 (28.6%)	15 (71.4 %)	
**Gender**			
Male	9 (78%)	30 (51.7%)	0.1
Body mass Index (> 30 kg/m^2^)	4 (33.3%)	8 (13.8%)	0.1
Diabetes	7 (58.3%)	16 (27.6%)	0.05
Hypertension	10 (83.3%)	37 (63.8%)	0.2
Cardiovascular diseases	1 (8.3%)	9 (15.5%)	0.5
Chronic obstructive pulmonary disease	2 (16.6%)	3 (5.1%)	0.2
Dyspnea	8 (66.7%)	13 (24%)	0.005
Impaired consciousness	6 (50%)	2 (3.4%)	0.00004
**Laboratory data**			
White cells count (x10^-9^/liter)	0.9 ± 0.3	0.6 ± 0.3	0.8
Lymphocyte count (x10^-9^/liter)	0.9 ± 0.4	1.50 ± 0.5	0.3
Aspartate aminotransferase (U/liter)	40 ± 58.1	31 ± 36.7	0.02
Alanine aminotransferase (U/liter)	24 ± 28.2	24.3 ± 32.2	0.6
**Thoracic CT scan**			0.1
Moderate	7 (58.3%)	45 (77.6%)	
Severe	5 (41.7%)	12 (20.7%)	
ICU hospitalization	4 (33.3%)	2 (3.4%)	0.000001
**Treatment**			0.3
Hydroxychloroquine	11 (91.7%)	44 (75.9%)	
Antiviral therapy	1 (8.3%)	13 (22.4%)	

ICU: intensive care unit

## Discussion

The SARS-CoV-2 infection has affected a significant number of people and has caused more than one million deaths to date worldwide. Epidemiological surveys have shown that the elderly or patients with comorbidities are more vulnerable to COVID-19, the incidence of severe cases and the risk of death were high. Hemodialysis centres are high-risk places, and hemodialysis patients are considered a vulnerable population during the COVID-19 pandemic [10]. The prognosis for hemodialysis patients with COVID-19 is still unclear, and more studies are needed. Among the 891 hemodialysis patients in the province of Blida, we collected 70 hemodialysis patients with SARS-CoV-2 disease confirmed by RT-PCR, which represents an incidence of 7.8% (the incidence of infection of hemodialysis patients in our centre was 12%). Alberici [11] noted an incidence of 15%, 36 hemodialysis patients (12.7%) were affected within one month in the Goicoechea series [12] and seven patients (3.5%) in the Wang study [13].

The mean age of our patients was 60.1 ± 15.0 years (18 - 88 years), of which 56.9% were men, 55.5% of patients were men with an average age of 61 years in Yiqiong's [10]. Obesity may be a risk factor for severe SARS-CoV-2 infection [14], in our study it did not have an impact on mortality [RR: 2.42; OR: 3.13(CI: 0.76 - 12.86); p = 0.1]. Symptoms in hemodialysis patients with COVID-19 infection were similar to those not on dialysis [15]. Our patients presented with asthenia (17.5%), fever (44.5%), cough (64.5%) and digestive disorders such as diarrhea in 4.7% of cases. Dyspnea was a symptom on admission in 30% of our patients, it was a predictor of mortality (OR = 6.9 [1.79-26.68]; p = 0.005), it represented (OR = 2.9 [1.24-7.07]; p = 0.014) in the Chawki study [16]. Lymphopenia was the most frequently encountered biological abnormality; it was present in 63% of our cohort and was 86% to 50% in some studies [17,18]. Leukopenia was present in 14% of Wang's patients [13] and 11% in Yiqiong's series [10]. Aspartate aminotransferase (AST) were elevated in 16.6% of patients in Wang's study [13] and 37% in Huang's series [17]. On admission, the pulmonary involvement by CT of our patients was moderate in 75% of cases and severe in 23.6%. In Wang's study [13], the involvement was bilateral in all patients with ground glass opacities and consolidation, Alberici [11] found bilateral involvement in 45% and unilateral in 25% of patients. Yiqiong [10] had found in his patient's bilateral involvement in 80% of cases with ground glass-opacities. Hydroxychloroquine has been prescribed in COVID-19 patients due to its action of blocking early transport of SARS-CoV-2 from endosomes to endolysosomes, which may be necessary for the release of the viral genome [19].

In the Maisonnasse primate study [20], the use of hydroxychloroquine with or without azithromycin did not reduce the viral load of the upper or lower respiratory tract, nor demonstrated clinical efficacy. Cavalcanti [21] in a randomized controlled trial, neither hydroxychloroquine alone nor hydroxychloroquine plus azithromycin improved clinical outcomes in hospitalized patients with mild to moderate COVID-19. However, Arshad [22] reported a survival benefit in hospitalized patients who received either hydroxychloroquine alone or hydroxychloroquine plus azithromycin, compared to those who received no medication. The prescription of different therapies (hydroxychloroquine and antibiotics) was not associated with a decrease in mortality (p = 0.9) in the Chawki cohort [16]. Alberici [11] reported that hydroxychloroquine was prescribed in 77% vs 20% for antivirals in hemodialysis patients. In the study of Cao [23]; treatment with Lopinavir- Ritonavir: did not significantly accelerate the time to clinical improvement (RR: 1.31; [95% CI]: 0.95 - 1.80), did not reduce mortality (19.2% vs 25%; [95% CI], -17.3 to 5.7) or did not decrease the detectability of throat viral RNA in patients with severe COVID-19 compared to a control group. In our cohort, there was no statistically significant difference from the treatment used (p = 0.3). One patient with minimal pulmonary involvement received the only azithromycin in our cohort, which was observed in 49% of Alberici patients [11].

Mortality was 17.1% in our series (mortality in the general population with COVID-19 disease is 3.5% in Algeria), occurring 8 ± 7.5 days (1 - 25 days) of hospitalization [in multivariate analysis, the factors associated with the risk of death were: dyspnea (p = 0.005), severe pulmonary involvement on CT (p = 0.03) and diabetes (p = 0.02)]. For their part, Chawki [16] and Goicoechea [12] reported 18% and 30% deaths, respectively. The mortality rate for Scarpioni [24] and Alberici [11] was 41% in Piacenza and 25% in Brescia (Italy) respectively. In Alsace, the most affected region of France, Keller [25] described a mortality of 24%, including the determinants associated with the risk of death (body temperature (HR 1.96; 95% CI 1.11 -3.44; p = 0.02) and elevated CRP at diagnosis (HR 1.01; 95% CI 1.005-1.017; p <0.0001); Hebibi [26] found 22% of deaths occurring between 3 and 5 days of hospitalization in hemodialysis patients treated in 3 hemodialysis centres. Yiqiong [10] in Wuhan (China) described six deaths (16.2%) in hemodialysis patients who tested positive for SARS- CoV-2, whose presumed causes of death were heart failure (2 cases), cerebrovascular involvement (1 case), hyperkalemia (2 cases) and the cause of death was undetermined in one patient.

**Limitations:** our study has certain limits. First, some laboratory parameters could not be achieved, such as serum ferritin; interleukin-6 and some were not performed in all patients. Second, we do not know the incidence of SARS-CoV-2 infection in hemodialysis patients because an RT-PCR test had only been performed in symptomatic patients.

## Conclusion

Mortality is higher in the hemodialysis population compared to the general population. It is essential to reduce the vulnerability of hemodialysis patients to future outbreaks of epidemics and reduce the risks to hemodialysis patients in the centre during dialysis sessions.

### What is known about this topic

- COVID-19 infection has not been sufficiently studied in this group of patients;
- To objectify the impact of COVID-19 infection on the hemodialysis population which is already vulnerable by renal failure.

### What this study adds

- Hemodialysis centres can be a site for the spread of infection due to the mobility of hemodialysis patients given the incidence of infections and mortality compared to the general population;
- No impact of hydroxychloroquine on the course of the infection;
- Share our modest experience with our African colleagues.
